# Postoperative, but not preoperative, inflammation-based prognostic markers are prognostic factors in stage III colorectal cancer patients

**DOI:** 10.1038/s41416-020-01189-6

**Published:** 2020-12-01

**Authors:** Kohei Yasui, Dai Shida, Yuya Nakamura, Yuka Ahiko, Shunsuke Tsukamoto, Yukihide Kanemitsu

**Affiliations:** 1 Department of Colorectal Surgery, National Cancer Center Hospital, 5-1-1 Tsukiji, Chuo-ku, Tokyo 1040045 Japan; 2 Division of Frontier Surgery, The Institute of Medical Science, The University of Tokyo, 4-6-1 Shirokanedai, Minato-ku, Tokyo 1088639 Japan

**Keywords:** Gastrointestinal cancer, Tumour biomarkers

## Abstract

**Background:**

Recent evidence suggests that both preoperative and postoperative inflammation-based prognostic markers are useful for predicting the survival of colorectal cancer (CRC) patients. However, associations between longitudinal changes in inflammation-based prognostic markers and prognosis are controversial.

**Methods:**

The subjects of this study were 568 patients with stage III CRC between 2008 and 2014. Preoperative and postoperative neutrophil-to-lymphocyte ratio (NLR), lymphocyte-to-monocyte ratio (LMR), C-reactive protein/albumin ratio (CAR) and lymphocyte-to-C-reactive protein ratio (LCR) were calculated to assess the inflammatory state of subjects. Subjects were stratified into three groups for each marker: preoperatively low inflammatory state (normal group), preoperatively high but postoperatively low inflammatory state (normalised group) and persistently high inflammatory state (elevated group). Multivariable analyses for overall survival (OS) and recurrence-free survival (RFS) were performed to adjust for well-established clinicopathologic factors.

**Results:**

For all assessed markers, the normalised group had a significantly better prognosis than the elevated group and a similar prognosis as the normal group for both OS and RFS.

**Conclusions:**

Postoperative, but not preoperative, inflammation-based prognostic markers more accurately predict OS and RFS in patients with stage III CRC.

## Background

Inflammation is considered a hallmark of cancer.^1^ Accumulating evidence suggests that the host inflammatory response plays an important role in the development and progression of cancer.^2^ Neutrophils, monocytes and C-reactive protein (CRP) reflect a systemic inflammatory response and are factors that contribute to cancer growth and spread.^3^ In contrast, lymphocytes and albumin reflect the immune and nutritional state of the host and provide information regarding a cancer patient’s capacity to mount a systemic inflammatory response.^4^ Various preoperative inflammation-based prognostic markers, such as neutrophil-to-lymphocyte ratio (NLR),^5–7^ lymphocyte-to-monocyte ratio (LMR),^8,9^ CRP/albumin ratio (CAR)^10–12^ and lymphocyte-to-CRP ratio (LCR),^3,4^ have been reported in previous studies to be prognostic for colorectal cancer (CRC). These studies suggest that a high level of preoperative systemic inflammation, as reflected by a high NLR, low LMR, high CAR and low LCR, is associated with worse outcomes in CRC patients.

The resection of a tumour can alter a patient’s inflammatory state since preoperative systemic inflammation in the cancer-bearing state might differ from postoperative systemic inflammation in the non-cancer-bearing state. Consistent with this, a previous study that examined longitudinal changes of NLR in CRC patients throughout the perioperative period found that postoperative NLR values on days 56–90 were slightly decreased relative to preoperative values.^13^ Associations between longitudinal changes in inflammation-based prognostic markers and patient prognosis are controversial, with some studies finding that the transition from a high preoperative inflammatory state to a low postoperative inflammatory state is associated with a good prognosis,^13,14^ and others finding no such association.^15^ Thus, investigating whether postoperative changes in various inflammation-based prognostic markers relative to preoperative levels are associated with survival differences in CRC patients undergoing resection is of particular interest.

Here, we hypothesised that postoperative systemic inflammation in a non-cancer-bearing state, rather than preoperative systemic inflammation in a cancer-bearing state, may reflect intrinsic host-related systemic inflammation, and thus normalisation of systemic inflammation after the surgery may improve survival. To test this, this study aimed to evaluate the prognostic impact of longitudinal changes in various inflammation-based prognostic markers in a large-scale stage III CRC population, with a particular focus on the transition from a high preoperative inflammatory state to a low postoperative inflammatory state.

## Methods

### Patient cohort

Subjects of this retrospective study were stage III CRC patients who underwent curative resection at the Department of Colorectal Surgery of the National Cancer Center Hospital between January 2008 and December 2014. Curative resection was defined as the lack of any gross residual cancer and no exposure of cancer cells in the surgical resection margin. Tumours were staged using the tumour-node-metastasis (TNM) staging system, 8th edition.^16^ Patients with anal canal cancer or appendiceal cancer, as well as those with tumours other than adenocarcinoma, were excluded from the cohort. Patients who did not visit for the first postoperative consultation within 21–90 days, or who had clinical evidence of an active infection or a chronic inflammatory condition at the postoperative visit, were excluded as described in a previous report.^17^ This study was approved by the Institutional Review Board (IRB) of the National Cancer Center Hospital (IRB code: 2017-437).

### Data collection and measurement of inflammation-based prognostic markers

Preoperative and postoperative blood test data were obtained from medical records. Preoperative data were defined as results from the final blood test performed before surgery. Postoperative blood samples were defined as those taken within 21–90 days after surgery but before starting adjuvant chemotherapy, since previous studies have reported that the level of systemic inflammatory response after surgery returned to preoperative levels from 21 to 90 days after surgery,^13^ and that adjuvant chemotherapy can lead to inconsistencies in blood test data due to adverse events such as cytopenia.^13^ The flow cytometry was used to count blood cells, the bromocresol purple method was used to quantify albumin, and the latex agglutination test was used to quantify CRP.

NLR was calculated as the absolute count of neutrophils (number/µL) divided by the absolute count of lymphocytes (number/µL).^18^ LMR was calculated as the absolute count of lymphocytes divided by the absolute count of monocytes (number/µL).^9^ CAR was calculated as levels of CRP (mg/dL) divided by those of albumin (g/dL).^10^ LCR was calculated as the absolute count of lymphocytes divided by CRP.^3^ High values of NLR and CAR, and low values of LMR and LCR, were considered to reflect a high inflammatory state. For each marker, patients were stratified into three groups: preoperatively low inflammatory state (normal group), preoperatively high but postoperatively low inflammatory state (normalised group) and a persistently high inflammatory state (elevated group). We analysed overall survival (OS) and recurrence-free survival (RFS) for each of these three groups. In subgroup analyses, we divided the normal group into a persistently normal group and exacerbation group to determine whether grouping them together into the normal group influenced the results.

### Follow-up

Postoperative follow-up consisted of serum CEA and CA19-9 measurements every 3 months for the first 3 years, then every 6 months for 3 years; computed tomography (CT) every 6 months for 5 years; and colonoscopy in the first and third year after surgery as described previously.^19^ Follow-up data were documented prospectively until an event occurred, or until the study cut-off date of November 2019.

### Statistical analysis

For each assessed marker, a single cut-off value calculated based on receiver-operating characteristic (ROC) curve analysis with reference to 5-year OS was used. The Wilcoxon signed-rank test was used to identify significant differences in preoperative and postoperative values of each marker. Scatter plots were generated, and correlation coefficients were calculated to investigate mutual relationships between each postoperative marker. The Kaplan–Meier method was used to estimate OS and RFS for each group. Differences in survival outcomes were assessed with the log-rank test and adjusted using Bonferroni correction. Multivariable Cox proportional hazards regression models were used to evaluate the relationship between longitudinal changes in inflammation-based prognostic markers and OS or RFS, adjusting for potential confounding covariates such as tumour site,^20^ preoperative CEA levels,^21^ histologic differentiation,^16^ T categories,^16^ N categories^16^ and use of adjuvant chemotherapy.^21^ Results are presented as hazard ratios (HRs) with 95% confidence intervals (CIs). Concordance index predicted by preoperative values, postoperative values, and longitudinal changes of markers was calculated. Time-dependent ROC curves were generated to incorporate time dependency of the disease state and each marker into the ROC curve approach, and integrated areas under the curves (integrated AUC) that is AUC through the results of time-dependent ROC curves at some points in time was also calculated. The prognostic impact of preoperative values and postoperative values and longitudinal changes were compared for each marker.^22,23^

All statistical analyses were performed using JMP version 14 (SAS Institute Japan Ltd., Tokyo, Japan) and R ver.3.6.0 (R Foundation for Statistical Computing, Vienna, Austria). The R package “timeROC” was used for time-dependent ROC analyses, and the package “survcomp” was used to calculate and compare the concordance index and integrated AUC. *P* < 0.05 was considered statistically significant for the Wilcoxon signed-rank test and multivariable analyses. For Kaplan–Meier analyses, *P* = 0.05 divided by the number of statistical tests (e.g., *P* < 0.017 if three tests (0.05/3 = 0.017) or *P* < 0.008 if six tests (0.05/6 = 0.008)) using Bonferroni’s correction was considered statistically significant.^24^

## Results

### Characteristics of the study cohort

Details of the study cohort are summarised in Supplementary Fig. 1. Between 2008 and 2014, a total of 636 stage III CRC patients who underwent curative resection at the National Cancer Center Hospital were identified. Of these, the following patients have excluded: 60 patients who lacked postoperative blood samples within 21–90 days after surgery, and 8 patients who had clinical evidence of an active infection or inflammation at the postoperative visit (three anastomotic leakages, one intraperitoneal abscess, one surgical site infection, one bowel obstruction, one incarcerated inguinal hernia and one acute subdural haematoma). Accordingly, the final study population consisted of 568 consecutive patients.

Patient characteristics are shown in Table 1. Postoperative NLR and CAR values were lower than their corresponding preoperative values, and postoperative LMR and LCR values were higher than their corresponding preoperative values. Changes in these markers postoperatively relative to preoperative values reflect a significant reduction in systemic inflammation after curative resection. The number of patients who received adjuvant chemotherapy was 450 (79.2%); 70 patients (15.6%) received oxaliplatin plus 5-fluorouracil (5FU), 376 patients (83.6%) received 5FU monotherapy and 4 patients (0.8%) received other chemotherapy. The median number of days between the preoperative blood test and surgery was 30 (interquartile range (IQR): 18–43) days, and that between surgery and the postoperative blood test was 41 (IQR: 33–55) days. Preoperative adjuvant chemoradiotherapy was performed in three patients with rectal cancer. Postoperative adjuvant chemoradiotherapy was performed only in one case because the standard treatment for stage III CRC in Japan is surgery and adjuvant chemotherapy.

**Table 1:** Baseline clinicopathologic characteristics of patients.

Clinicopathologic characteristics (*n* = 568)			
Age, years—median (range)	63 (28–92)		
*Sex,* n *(%)*			
Female	259 (45.6)		
Male	309 (54.4)		
*Site,* n *(%)*			
Colon	362 (63.7)		
Rectum	206 (36.3)		
*CEA,* n *(%)*			
<5 ng/ml	372 (65.5)		
≥5 ng/ml	196 (34.5)		
*Histologic differentiation,* n *(%)*			
Differentiated	529 (93.1)		
Others	39 (6.9)		
*T category,* n *(%)*			
pT1	51 (9.0)		
pT2	73 (12.9)		
pT3	365 (64.2)		
pT4	79 (13.9)		
*N category,* n *(%)*			
pN1	407 (71.7)		
pN2	161 (28.3)		
*Lymphatic involvement,* n *(%)*			
ly(+)	274 (48.2)		
ly(−)	294 (51.8)		
*Venous involvement,* n *(%)*			
v(+)	448 (78.9)		
v(−)	120 (21.1)		
*Adjuvant chemotherapy,* n *(%)*			
No	118 (20.8)		
Yes	450 (79.2)		
Oxaliplatin + 5FU	70 (15.6)		
5FU monotherapy	376 (83.6)		
Others	4 (0.8)		
Days between preoperative blood test and surgery, median (IQR)	30 (18–43)		
Days between surgery and postoperative blood test, median (IQR)	41 (33–55)		

	Preoperative	Postoperative	*P* value*
Albumin g/dl, median (IQR)	4.3 (4.0–4.5)	4.2 (3.9–4.4)	<0.001
CRP g/dl, median (IQR)	0.1 (0.05–0.25)	0.1 (0.04–0.2)	<0.001
Neutrophils/µl, median (IQR)	3793 (2941–4678)	3038 (2357–3871)	<0.001
Lymphocytes/µl, median (IQR)	1707 (1367–2038)	1706 (1392–2085)	0.06
Monocytes/µl, median (IQR)	280 (228–371)	278 (221–350)	0.12
NLR, median (IQR)	2.23 (1.70–3.96)	1.75 (1.32–2.33)	<0.001
LMR, median (IQR)	6.02 (4.54–7.70)	6.38 (4.95–7.70)	<0.001
CAR, median (IQR)	0.025 (0.012–0.061)	0.023 (0.010–0.051)	<0.001
LCR, median (IQR)	15051 (6151–32,456)	19605 (8559–41,805)	<0.001

*CEA* carcinoembryonic antigen, *5FU* 5-fluorouracil*, NLR* neutrophil-to-lymphocyte ratio, *LMR* lymphocyte-to-monocyte ratio, *CAR* C-reactive protein/albumin ratio, *LCR* lymphocyte-to-C-reactive protein ratio, *IQR* interquartile range*, CRP* C-reactive protein.

*P value calculated using the Wilcoxon signed-rank test.

### Relationship between the four inflammation-based prognostic markers for individual patients

We generated scatter plots and calculated correlation coefficients to assess relationships between the four postoperative inflammation-based prognostic markers in individual patients. CAR and LCR were negatively correlated with each other (*r* = −0.727, *P* < 0.001), while NLR and CAR (*r* = 0.300, *P* < 0.001), as well as LMR and LCR (*r* = 0.293, *P* < 0.001), were weakly positively correlated with each other (data not shown). Weakly negative correlations were observed between the following marker pairs: NLR and LMR (*r* = −0.397, *P* < 0.001), NLR and LCR (*r* = −0.323, *P* < 0.001), and LMR and CAR (*r* = −0.232, *P* < 0.001) (data not shown). These results suggest that four inflammation markers were roughly overlapping in each patient.

### OS by inflammation-based prognostic marker

**Fig. 1:**
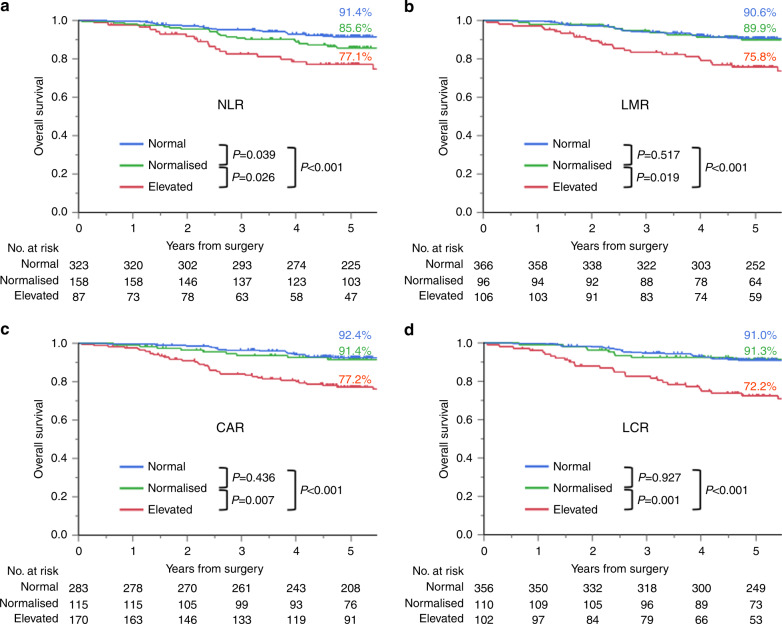
Overall survival curves for stage III colorectal cancer patients after curative resection by an inflammation-based prognostic marker (*n* = 568). Patients were stratified into three groups: preoperatively low inflammatory state (normal group), preoperatively high but postoperatively low inflammatory state (normalised group), and persistently high inflammatory state (elevated group). **a** NLR, **b** LMR, **c** CAR and **d** LCR. *P* < 0.017 was considered statistically significant.

ROC curve analyses yielded the following cut-off values: NLR (2.39), LMR (5.215), CAR (0.025) and LCR (10424). Figure 1 shows 5-year OS rates for the normal, normalised, and elevated groups by a marker (*P* < 0.017 using Bonferroni’s correction was considered significant in this analysis). For CAR and LCR, the normal group and normalised group had a significantly better prognosis for OS than the elevated group (CAR: *P* = 0.007, LCR: *P* < 0.001). For NLR and LMR, the prognosis for OS did not significantly differ between the normalised group and the elevated group (NLR: *P* = 0.026, LMR: *P* = 0.019). For all markers, no significant difference was observed in OS between the normal group and normalised group (NLR: *P* = 0.039, LMR: *P* = 0.517, CAR: *P* = 0.436, LCR: *P* = 0.927). The normal group showed a better prognosis than the elevated group for all markers (*P* < 0.001).

Postoperative blood tests were performed with a relatively wide range of periods (21–90 days after surgery). Therefore, the analysis was stratified into three groups according to the timing of the blood test. When the analysis was performed by postoperative blood test period (early group: 21–30 days, *n* = 105; middle group: 31–60 days, *n* = 372; late group: 61–90 days, *n* = 99), Kaplan–Meier curves for OS of these groups showed similar results to those of the entire cohort (data not shown). In addition, postoperative adjuvant chemotherapy is mandatory for patients with stage III colorectal cancer, and the clinical utility of this finding based on the receipt of adjuvant chemotherapy was also investigated.　We analysed the prognosis of patients receiving adjuvant chemotherapy (*n* = 450) as a subgroup. Kaplan–Meier curves for OS of the subgroup were similar to those of the entire cohort (data not shown).

To examine the validity of grouping the “persistently normal group” (inflammatory state stays low preoperatively and postoperatively) and “exacerbation group” (inflammatory state transitions from low preoperatively to high postoperatively) into the normal group, we conducted subgroup analyses for OS by dividing the normal group into the above-mentioned two groups (Supplementary Fig. 2; *P* < 0.008 using Bonferroni’s correction was considered significant in this analysis). The number of patients with exacerbation group for each marker was *n* = 43 for NLR, *n* = 63 for LMR, *n* = 79 for CAR and *n* = 64 for LCR. There was no significant difference in OS between the persistently normal group and exacerbation group for all assessed markers (NLR: *P* = 0.982, LMR: *P* = 0.934, CAR: *P* = 0.701, LCR: *P* = 0.231). This suggests that grouping the persistently normal group and exacerbation group together into the normal group was unlikely to impact the results. Accordingly, subsequent analyses were conducted with three groups (i.e., normal, normalised and elevated groups).

### Association between each inflammation group and OS

Multivariable analyses were performed for the four inflammation-based prognostic markers using Cox proportional hazards models (Table 2). After adjusting for known prognostic factors such as tumour site, preoperative CEA levels, histologic differentiation, T categories, N categories, and use of adjuvant chemotherapy, the normalised group had a significantly better prognosis for OS compared to the elevated group for all markers (NLR: HR, 0.49 (95% CI: 0.27–0.90), *P* = 0.022; LMR: HR, 0.42 (95% CI: 0.21–0.84), *P* = 0.014; CAR: HR, 0.44 (95% CI: 0.23–0.84), *P* = 0.013; LCR: HR, 0.35 (95% CI: 0.17–0.70), *P* = 0.003), and a similar prognosis as the normal group for all markers (NLR: HR, 1.43 (95% CI: 0.83–2.51), *P* = 0.198; LMR: HR, 0.93 (95% CI: 0.48–1.83), *P* = 0.850; CAR: HR, 0.89 (95% CI: 0.44–1.83), *P* = 0.759; LCR: HR, 0.70 (95% CI: 0.35–1.41), *P* = 0.318). The elevated group had a significantly poorer prognosis for OS compared to the normal group for all markers. Multivariable analyses without adjusting for the use of adjuvant chemotherapy also showed that the normalised group had a significantly better prognosis for OS compared to the elevated group for all assessed markers (NLR: HR, 0.48, (95% CI: 0.26–0.87), *P* = 0.016; LMR: HR, 0.40, (95% CI: 0.20–0.81), *P* = 0.011; CAR: HR, 0.44, (95% CI: 0.23–0.85), *P* = 0.014; LCR: HR, 0.35, (95% CI: 0.17–0.70), *P* = 0.003).

**Table 2:** Multivariable analysis of clinicopathologic variables in relation to overall survival by the inflammation-based prognostic marker.

	Multivariable analysis, HR (95% CI)							
Clinicopathologic variables	NLR	*P* value	LMR	*P* value	CAR	*P* value	LCR	*P* value
Site, rectum/colon	1.02 (0.62–1.71)	0.935	0.86 (0.53–1.42)	0.566	0.93 (0.56–1.54)	0.785	0.94 (0.57–1.55)	0.802
CEA (ng/ml), ≥5/<5	1.34 (0.83–2.16)	0.236	1.39 (0.86–2.23)	0.178	1.49 (0.92–2.41)	0.105	1.40 (0.86–2.28)	0.175
Histologic differentiation	2.65 (1.34–5.25)	0.005	2.74 (1.37–5.48)	0.004	2.43 (1.21–4.90)	0.013	2.33 (1.15–4.73)	0.019
*Others/differentiated*								
Tumour category, T4/T123	1.43 (0.78–2.63)	0.248	1.59 (0.87–2.89)	0.129	1.42 (0.78–2.61)	0.251	1.52 (0.83–2.80)	0.179
Nodal category, N2/N1	2.54 (1.58–4.09)	<0.001	2.54 (1.59–4.07)	<0.001	2.33 (1.45–3.74)	<0.001	2.27 (1.42–3.63)	<0.001
Adjuvant chemotherapy, no/yes	4.09 (2.56–6.55)	<0.001	3.91 (2.44–6.29)	<0.001	3.98 (2.46–6.43)	<0.001	4.04 (2.49–6.57)	<0.001
*Marker*								
Elevated/normal	2.91 (1.64–5.18)	<0.001	2.23 (1.33–3.74)	0.002	2.04 (1.21–3.48)	0.008	2.03 (1.20–3.41)	0.008
Normalised/elevated	0.49 (0.27–0.90)	0.022	0.42 (0.21–0.84)	0.014	0.44 (0.23–0.84)	0.013	0.35 (0.17–0.70)	0.003
Normalised/normal	1.43 (0.83–2.51)	0.198	0.93 (0.48–1.83)	0.850	0.89 (0.44–1.83)	0.759	0.70 (0.35–1.41)	0.318

*CEA* carcinoembryonic antigen, *NLR* neutrophil-to-lymphocyte ratio, *LMR* lymphocyte-to-monocyte ratio, *CAR* C-reactive protein/albumin ratio*, LCR* lymphocyte-to-C-reactive protein ratio.

### RFS by inflammation-based prognostic marker

**Fig. 2:**
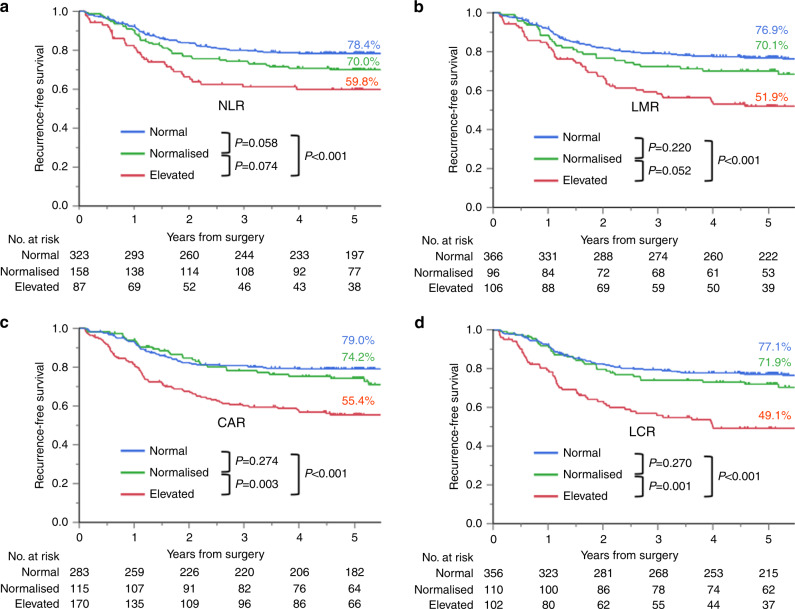
Recurrence-free survival curves for stage III colorectal cancer patients after curative resection by an inflammation-based prognostic marker (*n* = 568). Patients were stratified into three groups: preoperatively low inflammatory state (normal group), preoperatively high but postoperatively low inflammatory state (normalised group), and persistently high inflammatory state (elevated group). **a** NLR, **b** LMR, **c** CAR and **d** LCR. *P* < 0.017 was considered statistically significant.

Figure 2 shows RFS curves and 5-year RFS rates for the normal, normalised, and elevated groups by marker, with *P* < 0.017 considered significant for this analysis. For CAR and LCR, the normalised group had a significantly better prognosis for RFS than the elevated group (CAR: *P* = 0.003, LCR: *P* = 0.001). For NLR and LMR, the normalised group showed a non-significant trend of better prognosis for RFS compared to the elevated group (NLR: *P* = 0.074, LMR: *P* = 0.052). No significant differences were noted in RFS between the normal group and normalised group for all markers (NLR: *P* = 0.058, LMR: *P* = 0.220, CAR: *P* = 0.274, LCR: *P* = 0.270), whereas the normal group had a better prognosis for RFS than the elevated group for all markers (*P* < 0.001).

### Association between each inflammation group and RFS

Table 3 shows the results of multivariable analyses for RFS. After adjusting for the key clinical factors mentioned above, the normalised group had a significantly better prognosis for RFS compared to the elevated group for LMR, CAR and LCR (LMR: HR, 0.60 (95% CI 0.36–0.98), *P* = 0.041; CAR: HR, 0.59 (95% CI: 0.37–0.94), *P* = 0.025; LCR: HR, 0.59 (95% CI: 0.36–0.95), *P* = 0.030). The normalised group had a similar prognosis for RFS as the normal group for all markers (NLR: HR, 1.30 (95% CI: 0.89–1.91), *P* = 0.181; LMR: HR, 1.21 (95% CI: 0.77–1.92), *P* = 0.405; CAR: HR, 1.06 (95% CI: 0.66–1.70), *P* = 0.809; LCR: HR, 1.11 (95% CI: 0.72–1.73), *P* = 0.628). The elevated group had a significantly poorer prognosis for RFS compared to the normal group for all markers.

**Table 3:** Multivariable analysis of clinicopathologic variables in relation to recurrence-free survival by the inflammation-based prognostic marker.

	Multivariable analysis, HR (95% CI)							
Clinicopathologic variables	NLR	*P* value	LMR	*P* value	CAR	*P* value	LCR	*P* value
Site, rectum/colon	0.56 (0.40–0.80)	0.001	0.53 (0.38–0.74)	<0.001	0.55 (0.39–0.78)	<0.001	0.55 (0.39–0.77)	<0.001
CEA (ng/ml), ≥5/<5	1.42 (1.00–1.99)	0.046	1.43 (1.02–2.00)	0.037	1.49 (1.07–2.09)	0.019	1.47 (1.05–2.05)	0.024
Histologic differentiation	1.25 (0.66–2.35)	0.483	1.22 (0.65–2.31)	0.527	1.13 (0.60–2.13)	0.706	1.09 (0.57–2.05)	0.802
*Others/differentiated*								
Tumour category, T4/T123	1.32 (0.83–2.12)	0.244	1.40 (0.87–2.25)	0.162	1.33 (0.83–2.13)	0.234	1.35 (0.85–2.17)	0.199
Nodal category, N2/N1	2.20 (1.58–3.08)	<0.001	2.32 (1.66–3.24)	<0.001	2.10 (1.50–2.95)	<0.001	2.11 (1.50–2.95)	<0.001
Adjuvant chemotherapy, no/yes	2.03 (1.41–2.92)	<0.001	1.95 (1.36–2.83)	<0.001	1.94 (1.34–2.80)	<0.001	1.90 (1.31–2.75)	<0.001
*Marker*								
Elevated/normal	1.77 (1.16–2.71)	0.009	2.04 (1.39–2.99)	<0.001	1.79 (1.23–2.59)	0.002	1.90 (1.28–2.79)	<0.001
Normalised/elevated	0.73 (0.46–1.16)	0.187	0.60 (0.36–0.98)	0.041	0.59 (0.37–0.94)	0.025	0.59 (0.36–0.95)	0.030
Normalised/normal	1.30 (0.89–1.91)	0.181	1.21 (0.77–1.92)	0.405	1.06 (0.66–1.70)	0.809	1.11 (0.72–1.73)	0.628

*CEA* carcinoembryonic antigen, *NLR* neutrophil-to-lymphocyte ratio, *LMR* lymphocyte-to-monocyte ratio, *CAR* C-reactive protein/albumin ratio, *LCR* lymphocyte-to-C-reactive protein ratio.

### Recurrence and subsequent treatment patterns by marker

Information on recurrent and subsequent treatment patterns in patients in the normal and normalised groups were collected and is shown in Supplementary Table 1. For all markers, the normalised group had a higher recurrence rate than the normal group (NLR: 30 and 21%, LMR: 29 and 22%, CAR: 24 and 20%, LCR: 26 and 22%,). For the treatment of liver metastases, the normalised group had higher resection rates than the normal group for NLR (81 and 65%), LMR (88 and 68%) and LCR (72 and 70%). For lung metastasis, the normalised group had higher resection rates than the normal group for LMR (50 and 41%) and CAR (43 and 32%), and for local recurrence, the normalised group had higher resection rates than the normal group for NLR (50 and 29%), LMR (50 and 33%) and LCR (50 and 43%).

### Comparison of preoperative and longitudinal changes in inflammation-based prognostic markers for OS

**Fig. 3:**
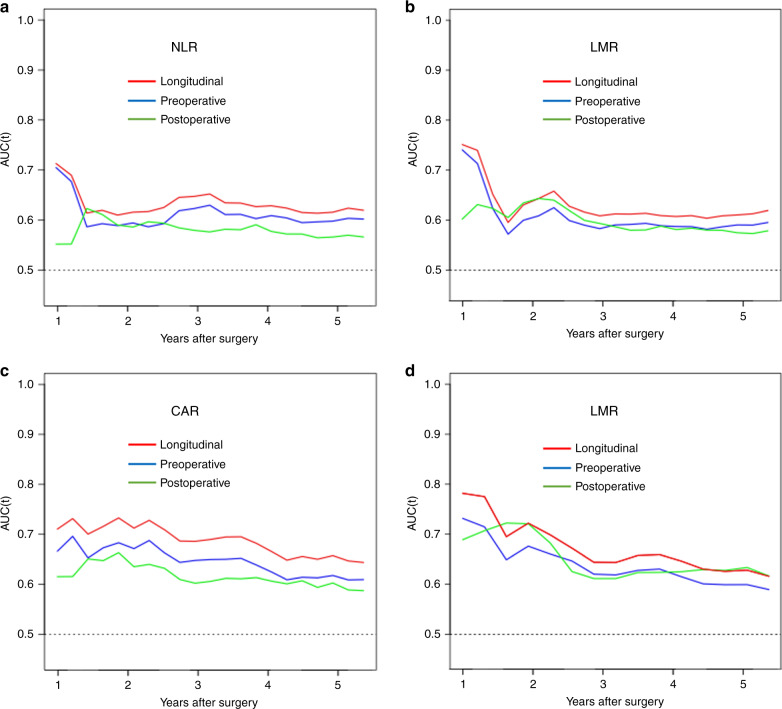
Time-dependent receiver-operating characteristic (ROC) curves for longitudinal changes and preoperative and postoperative inflammation-based prognostic markers. **a** NLR, **b** LMR, **c** CAR and **d** LCR.

Concordance index values and time-dependent ROC curves were used to compare the prognostic impact of preoperative inflammation-based prognostic markers and postoperative markers and longitudinal changes of markers for OS (Fig. 3). For all markers, concordance index values predicted by longitudinal changes of markers were higher than that predicted by preoperative markers (NLR: 0.691, standard error (SE) = 0.052 vs 0.683, SE = 0.043, *P* = 0.318, LMR: 0.675, SE = 0.047 vs 0.667, SE = 0.053, *P* = 0.751 CAR: 0.726, SE = 0.042 vs 0.723, SE = 0.051, *P* = 0.540, LCR: 0.742, SE = 0.046 vs 0.690, SE = 0.051, *P* = 0.823), and it was also higher than that of postoperative marker (NLR: 0.691, SE = 0.052 vs 0.663, SE = 0.056, *P* = 0.336, LMR: 0.675, SE = 0.047, vs 0.664, SE = 0.054, *P* = 0.414 CAR: 0.726, SE = 0.042 vs 0.685, SE = 0.052, *P* = 0.197, LCR: 0.742, SE = 0.046 vs 0.704, SE = 0.044, *P* = 0.796). No significant difference noted in any of the comparisons for concordance index values. Time-dependent ROC curves for longitudinal changes of inflammation-based prognostic markers were consistently superior to those of preoperative markers in all observation periods for NLR, LMR, CAR and LCR. Integrated AUC values of longitudinal changes (NLR: 0.640, LMR: 0.594, CAR: 0.598, LCR: 0.607) were significantly higher than those of preoperative markers (NLR: 0.611, LMR: 0.582, CAR: 0.577, LCR: 0.568) for all markers (*P* < 0.001). In addition, these were superior to those of postoperative markers in all observation periods for CAR and most observation periods for NLR, LMR and LCR. Integrated AUCs were also significantly higher than those of postoperative markers (NLR: 0.574, LMR: 0.578, CAR: 0.587, LCR: 0.589) for all markers (*P* < 0.001).

## Discussion

While some studies have evaluated longitudinal changes in systemic inflammation after surgery, to our knowledge this study is the first to focus on the prognostic impact of a normalised systemic inflammatory response after curative resection in stage III CRC patients. We found that longitudinal changes in inflammation-based prognostic markers such as NLR, LMR, CAR and LCR had a clear prognostic impact on stage III CRC patients undergoing curative resection. In support of this, Kaplan–Meier curves for OS showed that the normalised group had a significantly better prognosis than the elevated group and a similar prognosis as the normal group. Moreover, multivariable analyses for OS revealed that improvements in inflammation after surgery do not predict a worse prognosis. This suggests that even with a high preoperative inflammatory state, patients who transition to a low postoperative inflammatory state have a good prognosis for OS. In addition, the relationship between postoperative inflammatory state and prognosis was consistent regardless of the timing of blood sampling and the presence or absence of adjuvant chemotherapy.

Time-dependent ROC curve analyses further showed that curves for longitudinal changes of all markers were consistently superior to those for preoperative markers in all observation periods, and integrated AUC values predicted by longitudinal changes of markers were significantly higher than those of preoperative markers. Concordance index values predicted by longitudinal changes of markers were also higher than preoperative markers, although there was no significant difference. These results suggest that longitudinal changes of inflammation-based prognostic markers were more prognostically accurate than preoperative markers.

Results for RFS were similar to those for OS. Interestingly, while the OS curve of the normalised group was similar to that of the normal group, the RFS curve of the normalised group tended to be worse than that of the normal group, although not significantly so. The rate of resection of the recurrent site in the normalised group is higher than the normal group in some markers (Supplementary Table 1). Patients who underwent resection of the recurrent disease may have a good OS. The normalised group may have a higher recurrence resection rate than the normal group and good OS (similar to the normal group), while having a higher recurrence rate and poor RFS. The prognosis for OS and RFS did not significantly differ between the normalised group and the normal group for NLR and LMR, unlike CAR and LCR. In multivariable analyses for RFS, the normalised group had a significantly better prognosis compared to the elevated group for LMR, CAR and LCR. Concordance index values were higher for CAR and LCR than for NLR and LMR, suggesting that CAR and LCR may be more useful as inflammation-based prognostic markers in clinical practice.

In the entire cohort, median values of NLR and CAR decreased postoperatively while those of LMR and LCR increased relative to their preoperative values. These results are consistent with previous reports that assessed the postoperative host-related inflammatory state, in which systemic inflammation was found to decrease after surgery due to a reduction in tumour burden.^13,25^ On the other hand, the mechanism of persistent elevation of the systemic inflammatory response in CRC patients who underwent curative resection remains unclear. Some studies suggest that high inflammation levels indicate micro-metastatic disease or potential residual lesions,^13,15,17,26^ while others suggest that immunological homoeostatic decompensation by the surgical injury may be the cause of inflammation.^26^ We hypothesised that postoperative elevation of inflammation reflects a patient’s intrinsic inflammatory state. To test this, we assessed inflammation-based prognostic markers 5 years after surgery in patients who belonged to the elevated group for any marker, but who did not experience recurrence for 5 years (*n* = 180). Blood test data 5 years after surgery were available for 105 patients. Of these, 95 (91%) exhibited a high inflammatory state for at least one marker. Thus, most patients with a persistently high postoperative inflammatory state continued to be in a high inflammatory state for 5 years in the absence of recurrence, suggesting that the persistently high perioperative inflammatory state is not due to micro-metastases or potential residual lesions, but due to the patient’s intrinsic inflammatory state. Moreover, the results also suggest that postoperative systemic inflammation in the non-cancer-bearing state, rather than preoperative systemic inflammation in the cancer-bearing state, may reflect intrinsic host-related systemic inflammation independently of cancer-related inflammation.^27,28^

Regardless of the postoperative inflammatory state, those with a low preoperative inflammatory state were allocated to the normal group. This is because the exacerbation of inflammation soon after curative resection in stage III CRC patients appears to be a temporary reaction. To examine the validity of grouping the “persistently normal group” and “exacerbation group” into the normal group, we performed an analysis of OS with all four groups and found that the exacerbation group had a similar prognosis as the persistently normal group for all markers (Supplementary Fig. 2). Based on this, we considered it unlikely that grouping the persistently normal group together with the exacerbation group would impact the results. In addition, time-dependent ROC curves for longitudinal changes of inflammation-based prognostic markers were superior to those of postoperative markers. Concordance index values and integrated AUCs predicted by longitudinal changes of markers were higher than those of postoperative markers. These results may support the validity of evaluating in three groups based on longitudinal changes rather than the simple value of pre- and postoperative high or low inflammatory state.

Our findings suggest the importance of anti-inflammatory drug intervention to prevent recurrence after curative resection in CRC patients. Recently, non-steroidal anti-inflammatory drugs (NSAIDs), aspirin, and histamine-2-receptor antagonists have been shown to suppress host-related inflammatory responses and prevent cancer recurrence.^2,28,29^ Some studies found that oral aspirin may be effective as adjuvant therapy for CRC.^30,31^ However, the optimal target population for anti-inflammatory intervention remains unclear. For breast cancer, NSAIDs have been reported to reduce recurrence in patients with a high NLR.^32^ Our present findings suggest that patients with a postoperatively high inflammatory state may be the optimal target population for anti-inflammatory intervention along with chemotherapy, and that this intervention may apply across all types of cancer.

We also conducted a similar analysis on stage II CRC patients (*n* = 366) who underwent curative resection in our hospital. Kaplan–Meier curves were similar to those for stage III CRC patients, in that the normalised group had a significantly better prognosis for OS compared to the elevated group and a similar prognosis as the normal group for all markers. Differences in prognosis among groups for stage II CRC patients were not as robust as those for stage III CRC patients, given the relatively good prognosis for stage II CRC (data not shown). In the multivariable analysis, the normalised group had a significantly better prognosis than the elevated group (LMR: HR, 0.23, 95% CI: 0.09–0.63, *P* = 0.004; CAR: HR, 0.38, 95% CI: 0.17–0.86, *P* = 0.021; LCR: HR, 0.35, 95% CI: 0.16–0.78, *P* = 0.010). Therefore, longitudinal changes in inflammatory markers may also be relevant to stage II CRC patients and may help identify high-risk populations in need of postoperative adjuvant therapy.

This study has some limitations. First, this study was retrospective in design and included patients from a single institution. However, the study population was relatively large and homogenous in terms of the cancer stage. Second, the timing of preoperative and postoperative blood tests was not strictly consistent. The difference in the timing of blood sampling could have nonetheless had some impact on results relating to inflammation status. Large-scale prospective studies with a fixed blood test period will be needed to confirm our findings. Third, no data were available regarding the genetic makeup of the lesions, such as KRAS, BRAF and microsatellite instability state. Fourth, marker cut-off values were determined using data from only cancer patients, and not individuals without cancer. Nonetheless, since all four assessed inflammation-based prognostic markers can be calculated using data routinely available from blood tests, our findings suggest the possibility of using these markers in routine clinical practice.

In conclusion, after adjusting for key clinical factors, the normalised group had a significantly better prognosis for OS and RFS compared to the elevated group, and a similar prognosis as the normal group, for all assessed inflammation-based prognostic markers. Postoperative, but not preoperative, inflammation-based prognostic markers indicate intrinsic host-related inflammation in a non-cancer-bearing state after curative resection and may reflect the prognosis of patients with stage III CRC after curative resection.

## Supplementary information

Supplementary documents

## Data Availability

All data generated or analysed during this study are included in the paper.
