# Supplementary material for: Postoperative, but not preoperative, inflammation-based prognostic markers are prognostic factors in stage III colorectal cancer patients
Source: Br J Cancer. 2020 Dec 1;124(5):933–41. doi: 10.1038/s41416-020-01189-6 (PMC7921100; doi:10.1038/s41416-020-01189-6)
Supplement: Supplementary file 1 — Supplementary documents [file 41416_2020_1189_MOESM1_ESM.docx]

**
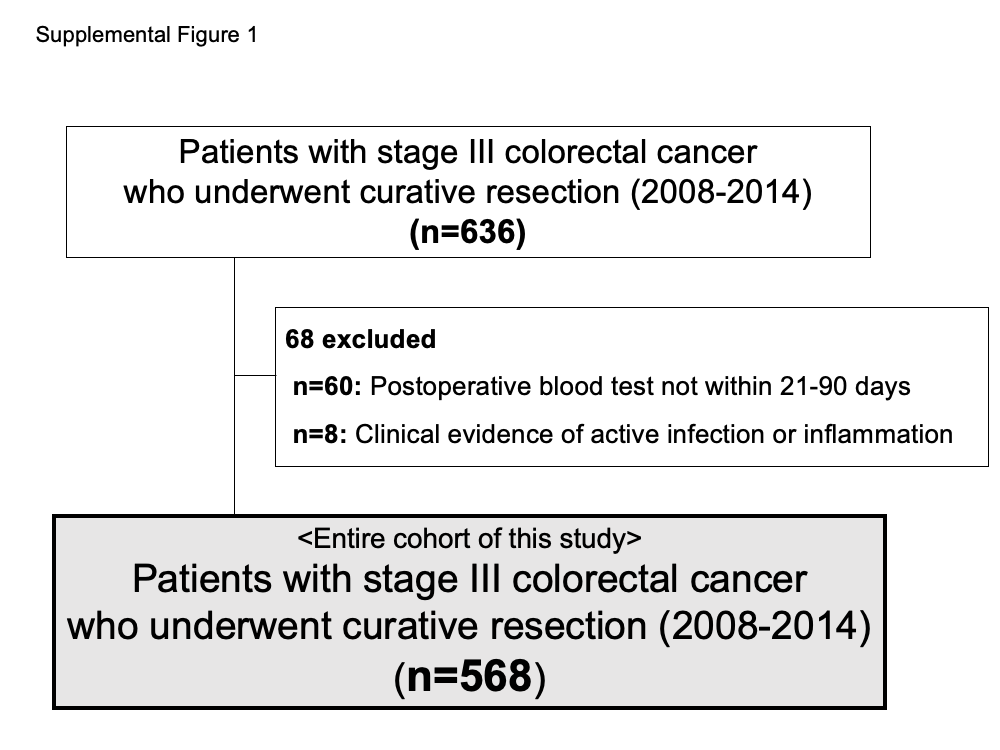
**

**
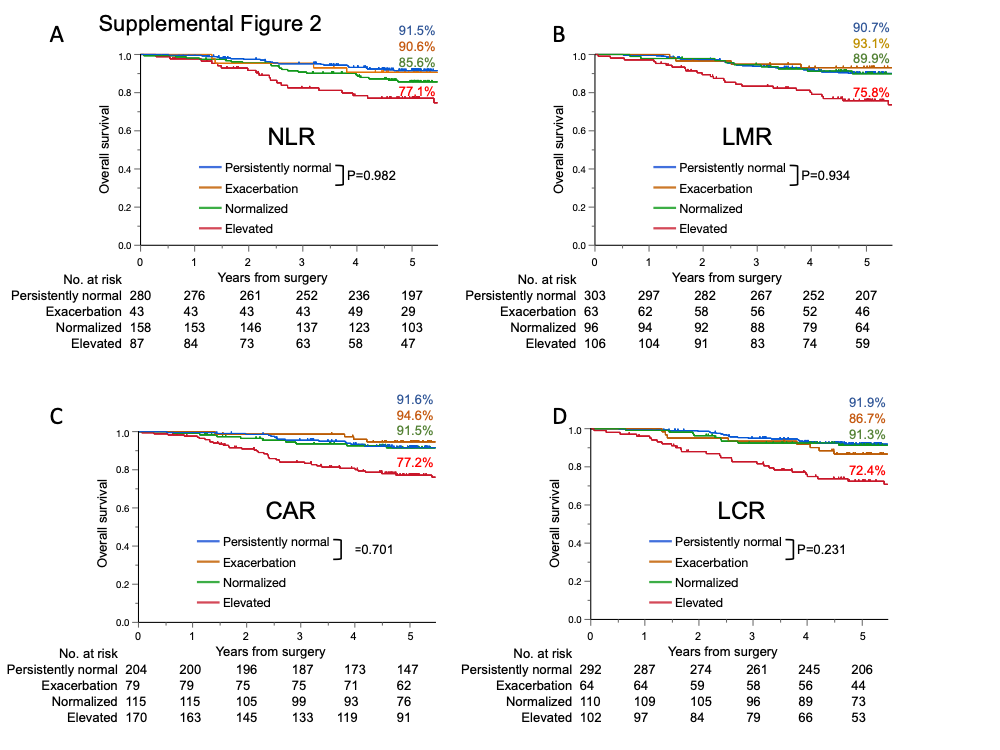
**

**SUPPLEMENTAL FIGURE LEGENDS**

Supplemental Figure 1. CONSORT diagram for patient selection.

After excluding patients who did not have their first postoperative blood test within 21-90 days of surgery (n=60), those whose infectious complications had persisted to the first postoperative visit (n=5), and those who had another disease at the first postoperative visit (n=3) from the initially recruited stage III colorectal cancer patients with curative resection (n=636), the final study population consisted of 568 patients.

Supplemental Figure 2. Overall survival curves for stage III colorectal cancer patients after curative resection by inflammation-based prognostic marker (n=568).

Patients were stratified into four groups: persistently normal group, exacerbation group, normalized group, and elevated group. (A) NLR, (B) LMR, (C) CAR, and (D) LCR. P<0.008 was considered statistically significant.
